# Long-term outcome for patients with distal radius fractures treated with volar locking plates versus percutaneous wires

**DOI:** 10.1371/journal.pone.0307763

**Published:** 2024-11-12

**Authors:** Linnea Wretö, Lotta Fornander

**Affiliations:** 1 Department of Orthopedic Surgery, Norrköping, Sweden; 2 Department of Biomedical and Clinical Sciences, Linköping University, Norrköping, Sweden; University Hospital Zurich, SWITZERLAND

## Abstract

**Background:**

Fractures of the distal radius are the most common fractures of the upper extremity. The choice of surgical method has been debated and studies show that short-term differences, in favour of volar locking plates, are indistinguishable at 1 year follow-up. Few studies have investigated long-term outcomes beyond one year. The aim of this study was to investigate long-term (6–10 years) patient-reported outcomes after distal radius fracture surgery, and to determine how fracture pattern/complexity (Buttazzoni fracture type) affects the results.

**Methods:**

303 patients surgically treated for a distal radius fracture, from 2012–2016 were included. The questionnaires used were PRWE, *Quick*-DASH and EQ-5D. Previous studies have shown that fracture type influences the choice of surgical method and therefore fracture type was adjusted for in the statistical analysis.

**Results:**

We found that patients treated with K-wires had significantly better Quick-DASH scores (*p* <0.05) and a lower degree of pain (PRWE pain scale) (*p* <0.05) than patients treated with volar locking plates. There was no difference between the two groups regarding the remaining outcome measures for any Buttazzoni type of fracture.

**Conclusion:**

On the basis of these results, K-wires can be considered an equivalent option to locking plates because of equal or better long-term outcomes, lower costs, and shorter operation times.

## Introduction

Fractures of the distal radius (DRF) are the most common fractures of the upper extremity, and in Sweden about 20,000 DRFs are reported every year [1]. Most patients with DRFs are treated non-surgically (74%) with repositioning and plaster casts. A smaller group (26%) does not achieve acceptable reduction after repositioning and therefore requires surgical treatment [2].

The choice of surgical method has been debated. Many studies have investigated the matter, however, the results are not concurrent [3–7], yet volar locking plates (VLP) have become the routine procedure in Sweden [8, 9], with a frequency rate of 85% compared to percutaneous kirchner wires (PKW), at 10% [10]. The reason for this development is not fully known but could possibly be attributed to the impact of the VLP industry, preferences of the individual surgeons, local traditions, or possible actual differences in outcome that are not yet proven. There are studies showing better results for patients treated with VLP regarding functionality—both self-reported and objectively measured—in the short term; up to 6 months after surgery compared to patients treated with PKW/External fixator (EF). However, the differences were indistinguishable at 1 year follow-up [3, 11, 12], and few studies with large patient cohorts comparing PKW and VLP have investigated long-term outcomes beyond one year. Because of the low correlation between radiological outcome and the results in terms of function and residual impairments [13] patient reported outcome measures were considered the most appropriate variable to evaluate.

There is only one multi-centre, large scale study made to this day comparing VLP and PKW; the UK DRAFFT, an RCT study including 461 DRF patients randomised to VLP or PKW treatment, between the years 2011–2012 [14]. The DRAFFT study found no difference between the treatment groups in terms of wrist function, pain, quality of life, or complications. Surgery with PKW was associated with significantly shorter operation time and lower overall costs. This suggests that PKW may be a more suitable option, given that the methods render similar outcomes. In the DRAFFT study, patients were followed up annually and after 5 years, a second follow-up study demonstrated there was still no significant difference in function, pain, or life quality [15].

To our knowledge, there are no previous studies presenting follow-up results longer than 5 years after DRF surgery on larger populations. Also, there are still too few studies investigating VLP versus PKW specifically to concurrently conclude which method is preferable for certain types of fracture patterns, with respect to outcomes such as function, pain, and quality of life.

The main aim of this study was to investigate long-term post-operative function, pain, and health-related quality of life, using patient-related outcome measures (PROM), in patients surgically treated for DRF with VLP or PKW. Our secondary aim was to determine the effect of the fracture pattern on the results.

## Methods

### Participants

This study is a continuation of an earlier retrospective cohort study [16], which investigated 346 patients surgically treated with either VLP or PKW for a DRF between 2012–2016. The aim of that study was to compare the surgical method and Buttazzoni type of fracture with functional outcome (range of motion and grip strength), duration, and frequentness of rehabilitation by reviewing the patient’s medical records.

**Fig 1:**
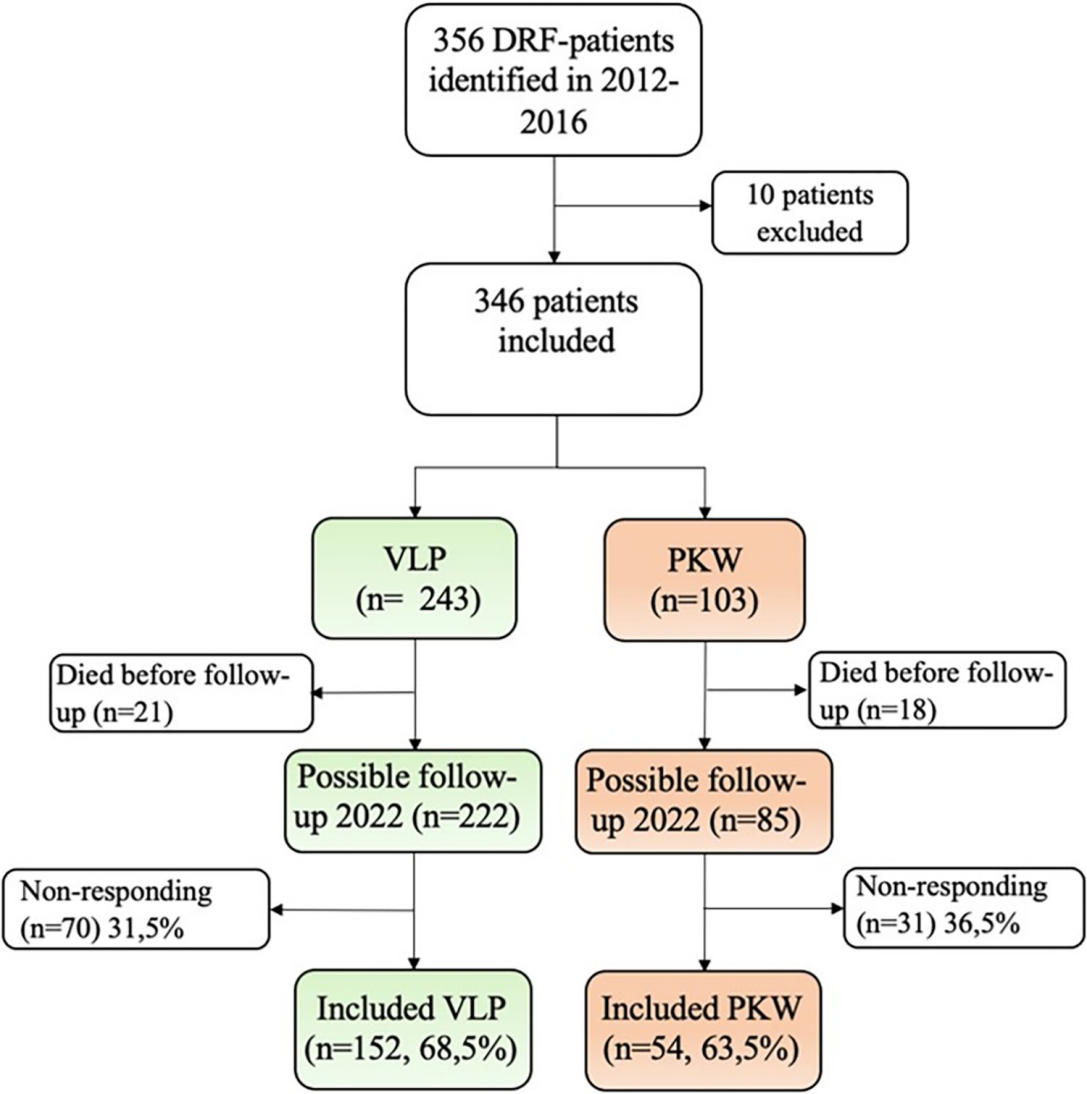
Flowchart of patients, from retrospective inclusion in 2021 to follow-up inclusion in 2022, divided by operation methods. Percentages in included treatment groups are relative to total possible inclusion in each group.

Original inclusion criteria made all adult patients over the age of 20 years (to exclude pediatric fractures) with a DRF treated with either VLP or PKW in 2012–2016 eligible for inclusion. Patients with missing prereduction radiographs, aged below 20 years, and patients who sustained bilateral DRFs within 30 days were excluded. Three hundred and fifty-six patients were eligible for inclusion and after examination according to exclusion criteria, and 346 patients were included in the previous study [16] (Fig 1). Using the same population, this study aims to follow up patient-related outcome measures (PROM) 6–10 years after surgery. Of the 346, 40 patients had died and were therefore not eligible for inclusion in this long-term follow-up. The possible number of participants eligible for inclusion was 306 and were grouped as shown in Fig 1.

DRFs are most common among post-menopausal women >50 years of age, the cause of injury is most often a low-energy trauma [2], and they are likely to have either incipient or concurrent osteoporosis at the time they fractured their wrist [17]. Patients, especially women, below 55 years are less likely to have osteoporosis, patients 55–69 years old are at increased risk to develop osteoporosis and patients from the age of 70 years generally have lower functional requirements.

Therefore, age groups (<55, 55–69, and ≥70 years) are used as an attempt to compensate for the distorted distribution of age and sex.

### Treatments, postoperative protocol, and rehabilitation

The choice of surgical method was made by the surgeon. VLP was generally preferred for younger patients and for fractures with higher grades of instability, as shown in a previous study [16], however both surgical methods were used for all Buttazzoni fracture types.

- Percutaneous wires: 2–4 wires were introduced through the skin to the fracture gaps on the dorsal and radial aspects of the radius, by hand, according to the Kapandji technique. The wires were used for anatomical reduction and were fastened by machine at the opposing cortex. Wires and plaster casts were removed 5 weeks postoperatively.
- Volar locking plate: Surgery was performed through a modified Henry approach for open reduction and plate application. The plate was attached to the volar aspect of the radius by fixed-angle screws in the distal part of the plate and non-locking screws in the shaft. Plaster casts were removed 2 weeks after the operation.

All patients were offered guided rehabilitation with an occupational therapist specialized in hand surgery and all patients participated in rehabilitation. The postoperative regimen and rehabilitation process followed 2 different tracks depending on the method of surgery. For patients treated with PKW the cast was removed five weeks postoperatively and a wrist orthosis was provided for use during sleep and activities. Mobility training was performed 6 times/day and patients were allowed full unloaded range of motion. For patients treated with VLP the cast was removed at 2 weeks postoperatively and a wrist orthosis was provided for continuous use. Mobility training was performed 6 times/d. At 5 weeks the wrist orthosis was used only during sleep and activities and full unloaded range of motion was allowed. The occupational therapist decided when the patient’s wrist function was sufficiently rehabilitated and terminated treatment at that time. Full weight bearing was allowed eight weeks after surgery.

### Measures

The PROMs selected in this study were patient-related wrist evaluation (PRWE), *Quick*-disability of the arm, shoulder, and hand (*Quick-*DASH), and EQ-5D. PRWE is a wrist-specific measure of function and pain [18]. It is a 15-item questionnaire divided into two subscales: pain, containing 5 items; and function, containing 10 items. Each question ranges from 0–10, where 0 corresponds to no pain or no difficulty, and 10 corresponds to the worst pain possible or unable to perform. The pain score is comprised of the 5 pain-items summed up, where 0 is the best score and 50 is the worst. The function score is the sum of the 10 function items divided by 2. The total score is the sum of the pain and function scores, ranging from 0—an adequately functioning wrist without pain—to 100, a totally disabled and very painful wrist. The shortened version of the DASH questionnaire was also used to measure functional outcome and symptoms [19]. It consists of 11 items, scored 1 (no difficulty) to 5 (unable to perform) and the total score is calculated with an equation and the total score ranges from 0–100. The EQ-5D-5L was used [20] to evaluate the quality of life. The questionnaire consists of 5 items: mobility, self-care, usual activities, pain/discomfort, and anxiety/depression, and each item is scored from level 1 (no problem) to level 5 (unable to perform). The scores of all items are then summed up and called the EQ-5D_index_, a scale ranging from 5 points (no problems) to 25 points (totally unable to perform). The EQ-5D also contains a EQ-VAS (visual analogue scale) score, where the responder rates their general health from 0 (worst health imaginable) to 100 (best health imaginable) [20]. Validated, translated versions (Swedish) of the PRWE [21, 22], *Quick*-DASH [23] and EQ-5D [20] were used in this study. Questionnaires were distributed on 1 April 2022 and non-responders received a reminder in June 2022.

### Fracture classification

The AO classification [24] is often used to classify DRFs but it has been criticised as lacking intra- and interobserver reliability [25, 26]. Additionally, the AO classification fails to adequately prove prediction of radiographic and clinical outcomes [27]. Therefore, in this study we instead used the Buttazzoni classification system, presented in 2009 as a development of Older’s classification of DRFs, which aims to describe fracture instability to anticipate the risk of fracture collapse, and suggest a treatment accordingly. It consists of five types of fracture patterns without subgroups (Table 1), and is the only classification system which takes the comminution of the volar cortex into consideration [28]. Furthermore, the Buttazzoni classification has shown fair-to-moderate to substantial intraobserver reproducibility and moderate interobserver reliability [28], compared to AO with fair-to-moderate reproducibility and fair interobserver reliability [25, 26]. The classification was performed, retrospectively, by a junior researcher supervised and verified by a senior consultant in Orthopedic surgery. Classification was performed based on conventional radiographs.

**Table 1:** The Buttazzoni classification of distal radius fractures.

◊ Buttazzoni 1: Extraarticular DRF with no cortical (metaphyseal) comminution.
◊ Buttazzoni 2: Extraarticular DRF with comminution of the dorsal cortex.
◊ Buttazzoni 3: Intraarticular (radio-carpal joint) DRF with or without metaphyseal comminution (completely articular fractures as in AO classification type C).
◊ Buttazzoni 4: DRF comminution of the volar cortex regardless of other coexisting fracture lines.
◊ Buttazzoni 0: DRF which cannot be classified according to the above types such as intraarticular fractures without metaphyseal comminution (partially articular fractures), e.g., carpal fracture-dislocation, Barton fractures and Chauffeur fractures.

### Ethical approval

This study was approved by the Swedish ethical approval authority (reference number 2021-06857-02). Patients gave informed written consent to participate in the study by returning the questionnaires. Pseudonymisation of all patients was performed for further data processing.

### Statistical analysis

Missing data was managed according to the respective questionnaire manual. All collected data was self-reported but normally distributed. Both parametric and non-parametric tests were executed but resulted in no significant differences, and the results of the parametric tests are presented. For analysis of the questionnaire results, independent T-tests were used. Grouping by surgical method and stratifying for Buttazzoni classification was performed. The Univariate general linear model (GLM) was used for further analysis of the independent variables of age and gender. Results were considered statistically significant for *p-*values <0,05. IBM SPSS Statistical program version 28.0.0 was used for statistical analysis.

## Results

### Demographics

The number of responding patients was 209, as 98 patients did not respond at all. Of the responding questionnaires three were excluded; (Fig 1) one due to concurrent severe polyneuropathy, one due to changed address, and one because of too-poor eyesight. In total, questionnaires from 206 patients (67.1% of possible follow-ups) were included for further analysis.

**Fig 2:**
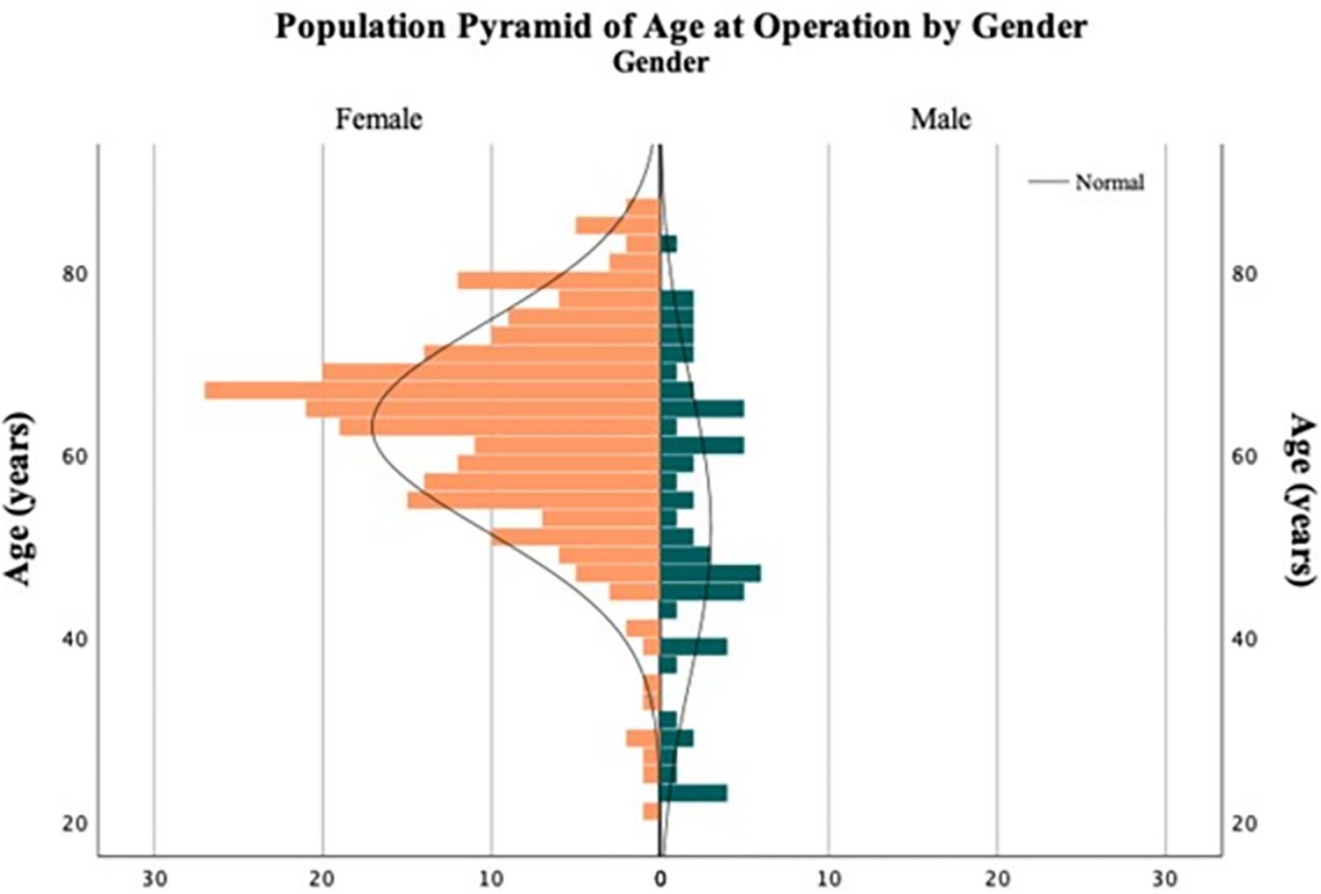
Age distribution. Age distribution of included females (n = 243) and males (n = 60).

The included females had a higher mean age (60.3 years) than the males (51.8 years) (*p* <0.001), (Fig 2). There was no difference (*p* = 0.756) in mean age between responding patients and non-responding patients. However, out of the responding patients, the PKW group had a higher mean age (66.5 years) than the VLP group (59.1 years) (*p* <0.001).

When analysing fracture pattern and complexity, there were too few subjects in Buttazzoni groups 0 and 1, and they were therefore not included in any further analyses (Table 2). There was no difference in mean age between the Buttazzoni groups, and no age difference between respondents and non-respondents within the Buttazzoni groups B2, B3 and B4.The total number of patients in the PKW group was 54 and in the VLP group 152 (Table 2).

**Table 2:** Number of patients included sorted by operation method and Buttazzoni type of fracture.

Operation method	Buttazzoni type	*N*
**PKW**		54
	**B0**	0
	**B1**	3
	**B2**	21
	**B3**	14
	**B4**	16
**VLP**		152
	**B0**	1
	**B1**	0
	**B2**	39
	**B3**	64
	**B4**	48
**Total**		206

### Outcome

**Fig 3:**
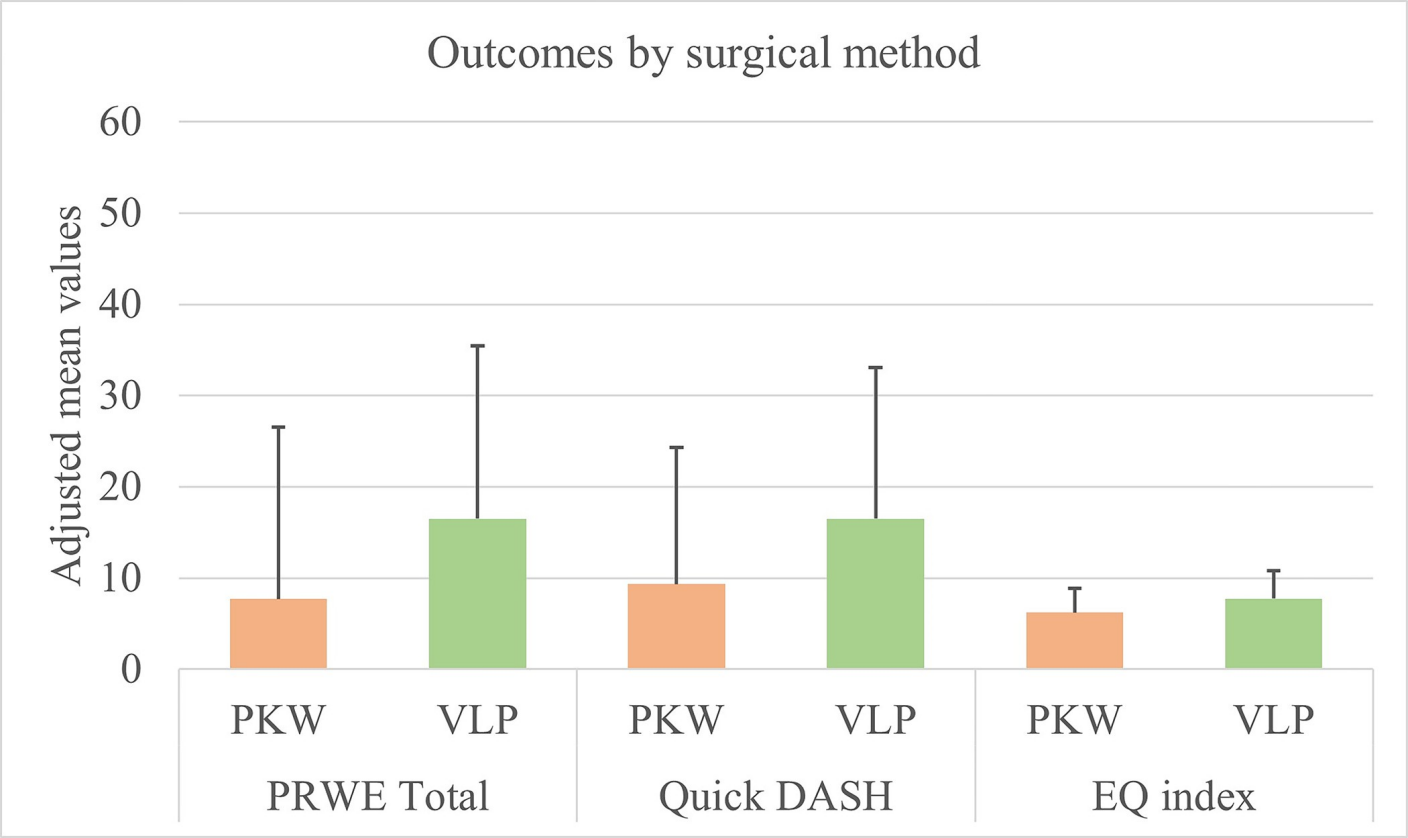
Adjusted mean values of PRWE total score, Quick DASH and EQ index grouped by operation methods PKW and VLP.

Independent T-tests revealed no significant differences between the surgical methods in any of the outcome measures. To adjust for confounding factors, age, sex and Buttazzoni type of fracture, further analysis with Univariate General Linear Model (GLM) was conducted. GLM analysis revealed that age, sex, and Buttazzoni type of fracture affected the results, and after adjustment, patients treated with PKW had significantly lower PRWE pain scores (p = 0.045) than patients treated with VLP (Table 3). When adjusting only for age, by the three patient age groups (<50, 50–69, and >69 years), patients in the PKW group had lower *Quick*-DASH scores than patients in the VLP group (p = 0.040). Moreover, patients in the PKW group had marginally lower total PRWE scores (p = 0.054) than those in the VLP group (Fig 3). However, there was no difference between the surgical methods in PRWE function score, EQ-5D_index_, or EQ-VAS (Table 3).

**Table 3:** Patient reported outcome measures.

Outcomes	PKW adj. Mean (SD)	VLP adj. Mean (SD)	95% CI of difference	p-value raw	P-value adj.
***Quick-*DASH**	9,31(±14,98)	16,36(±16,56)	0,341 to 13,76	0,566	0,040^b^*
**PRWE Pain score**	4,23(±10,0)	9,39(±10,94)	-0,494 to 10,81	0,294	0,045^a^*
**PRWE Function score**	3,2(±9,6)	7,30(±9,11)	-0,955 to 8,96	0,723	0,078^a^
**PRWE Total score**	7,68(±18,82)	16,5(±18,97)	-1,24 to 18,87	0,516	0,054^a^
**EQ-5D_index_**	6,19(±2,64)	7,72(±3,05)	-1,10 to 0,32	0,196	0,065^a^
**EQ VAS**	81,8(±19,48)	74,93(±18,27)	-0,84 to 14,58	0,701	0,077^b^

*p-value <0,05

^a^*p*-value from General Linear Model, adjusted for age group, Buttazzoni type and sex.

^b^*p*-value from General Linear Model adjusted for only age group.

Outcomes of Quick-DASH, PRWE, and EQ-5D 6–10 years after surgery, comparing operation methods, adjusted for confounders. Raw p-value is from independent T-test.

When comparing PKW and VLP groups stratified by fracture pattern/complexity—i.e., Buttazzoni types of fractures—there were no differences in the outcomes of *Quick*-DASH, PRWE, or EQ-VAS (Table 4). As for the EQ-5D_index_, there were no differences between the operation methods in Buttazzoni groups 2 or 3, regardless of adjustment for confounding factors. However, for the Buttazzoni 4 group, patients treated with PKW (adj. mean 5.97 ± 1.93) had significantly lower EQ-5D_indices_ than patients treated with VLP (adj. mean 8.5 ± 2.92) (*p* = 0.006).

**Table 4:** Patient reported outcome measures in relation to surgical methods and fracture type.

Outcomes	PKW Adj. Mean (SD)	VLP adj. Mean (SD)	95% CI of Difference	p-value Raw	p-value Adjusted
***Quick*-DASH**					
** B2**	5,72 (±14,90)	9,93 (±12,06)	-13,42 to 21,85	0,969	0,632^a^
** B3**	6,36 (±15,33)	16,39 (±12,36)	-12,17 to 32,25	0,415	0,370^a^
** B4**	9,11 (±15,56)	21,23 (±20,44)	-0,54 to 24,79	0,336	0,060^b^
**PRWE Total**					
** B2**	5,15 (±19,42)	11,82 (±15,37)	-15,97 to 29,32	0,891	0,556^a^
** B3**	1,5 (±15,96)	16,09 (±17,14)	-10,51 to 39,68	0,995	0,250^a^
** B4**	9,73 (±22,13)	22,84 (±13,34)	-1,75 to 27,97	0,524	0,083^b^
**EQ-5D_index_**					
** B2**	6,89 (±3,05)	7,23 (±3,48)	-4,61 to 5,28	0,583	0,892^a^
** B3**	5,5 (±2,88)	7,68 (±2,92)	-1,96 to 6,34	0,908	0,296^a^
** B4**	5,97 (±1,93)	8,50 (±2,92)	0,74 to 4,32	0,078	0,006^b^*
**EQ VAS**					
** B2**	89,32 (±19,20)	73,39 (±20,27)	-13,68 to 45,54	0,158	0,285^a^
** B3**	72,18 (±24,87)	76,41 (±18,84)	-24,04 to 32,5	0,142	0,766^a^
** B4**	81,44 (±13,82)	74,62 (±15,74)	-5,26 to 18,9	0,442	0,263^a^

*p-value <0,05

^a^*p*-value from General Linear Model, adjusted for age group, Buttazzoni type and sex.

^b^*p*-value from General Linear Model adjusted for only age group.

Outcomes of Quick-DASH and PRWE comparing surgical methods in Buttazzoni classes B2 through B4, adjusted for confounders. Raw p-value is from independent T-test.

## Discussion

Long-term patient-related outcome measures of distal radius fracture surgery comparing PKW and VLP have not previously been investigated for longer than five years after surgery. This study investigated patient-related outcome measures in 206 patients 6 to 10 years after surgery and found that patients treated with PKW had higher self-reported function, higher health-related quality of life, and a lower degree of pain than patients treated with VLP.

It can be argued that the differences we found, although statistically significant, are too small to imply clinical importance. Minimal clinically important difference has been set to 11.5–14 points for the PRWE and 14 points for the *Quick*-DASH [29] in a population of this size, and our results do not reach these levels. Nevertheless, long-term results showing no difference between PKW and VLP are not to be underestimated.

To further refine the investigation and to determine the preferred surgical method for specific fracture patterns, Buttazzoni types were stratified (by type), however, no difference was found in either PRWE or *Quick*-DASH between surgical methods for Buttazzoni groups 2–4. A difference in EQ-5D_indices_ was found only for the Buttazzoni 4 group, with better quality of life for patients treated with PKW. Wadsten et al. described that distal radius fractures with volar comminution (Buttazzoni 4) are prone to redislocate when treated non-surgically with a plaster cast [30]. Therefore, the more stable surgical method, VLP, is recommended by the Swedish national system of knowledge management of healthcare for the more complicated B3 and B4 fractures [1]. In the present study Buttazzoni fracture type did not predict the long-term outcome of different surgical methods.

In the earlier study conducted on our population [16], no short-term clinically important differences were found between the two surgical methods in functional outcome, measured by range of motion and grip strength. This was true for all included Buttazzoni fracture types (B2–B4) which leads us to the conclusion that although surgeons often prefer VLP for more complicated fractures, PKW fixation could be considered a surgical method for all Buttazzoni fracture types. Our results suggest that this argument is also valid regarding long-term patient-reported outcome.

Previous studies recommend VLP when fast recovery is required [3, 5–7, 12, 31], but long-term investigations have been inconclusive. Studies comparing locking plates and PKW (or EF with PKW) are often conducted on small populations and/or with short follow-up times [3, 32], which makes their results less reliable. Studies with a population of more than 100 patients have concluded PKW/EF is either superior to locking plates or has equal outcomes [4, 5, 7, 15, 16]. Moreover, few studies have investigated outcomes for longer than a year after surgery, but their conclusions are relatively concordant; PKW/EF is equal [4, 15] to surgery with VLP. These conclusions are in accordance with our results.

A possible confounding factor when investigating functional outcome after DRF is hand dominance. Fractures of the dominant hand may have a greater impact on the long-term functional outcomes of the wrist, and even on the patient’s quality of life. Unfortunately, there was not enough data available on hand dominance in our population to further investigate this, which is a limitation of this study.

Another limitation of the study is its cross-sectional design, thus preventing the possibility of preoperative comparison to continuous follow-up of PROMs. Also related to the cross-sectional design is an inability to form equal study groups. The VLP group is almost three times as large as the PKW group, and the PKW group has a higher mean age than the VLP group. However, the patients were retrospectively included over five years, possibly making the overall population larger than what would have been possible for an RCT with a similar purpose, and the age difference was adjusted for during the regression analysis. The DRAFFT study is a prospective randomized study of high quality, however fractures where the articular surface could not be repositioned by indirect techniques were excluded, which makes the results less generalizable for intraarticular fractures with misalignment. In the present study, fractures of all Buttazzoni types were treated with both methods of surgery and our results remain concordant with the results of the DRAFFT study.

An obvious confounding factor in this type of comparison is fracture pattern. It is commonly held that volar plate fixation is chosen for fractures with greater complexity, and this perception has been proven true in an earlier study by our group [16]. Because of this, fractures were classified according to the Buttazzoni classification and type of fracture was adjusted for in the regression analysis.

The strength of this study is that it included a larger population than most studies on the subject, and the response rate was quite high, at 67%, even though many years had passed since surgery.

## Conclusion

In conclusion, we found significant differences in self-reported functional outcome, pain, and health-related quality of life in favour of PKW, 6 to 10 years after surgery. Although the clinical relevance of these differences may be disputable, the non-inferiority of PKW is an important result, since surgery with PKW is a cost-saving alternative and associated with shorter operation time.

## Supporting information

S1 Data Raw data file containing Quickdash (qD), EQ5D (E) and PRWE(P) answers, method of surgery (op_method), fracture classification (Buttazzoni), gender (sex) and age at surgery (ageatop). (XLSX)
